# Universality without uniformity – infants’ reactions to unresponsive partners in urban Germany and rural Ecuador

**DOI:** 10.3758/s13421-022-01318-x

**Published:** 2022-05-10

**Authors:** Helen Wefers, Nils Schuhmacher, Ledys Hernández Chacón, Joscha Kärtner

**Affiliations:** 1 Department of Psychology, University of Münster, Fliednerstraße 21, 48149 Münster, Germany; 2 Department of Educational Sciences, University of Otavalo, Otavalo, Ecuador

**Keywords:** Still-face effect, No-touch effect, Parenting styles, Early social expectations

## Abstract

Previous studies based on non-WEIRD (Western, Educated, Industrialized, Rich, and Democratic) samples provide initial evidence that the still-face effect is universal. Based on the assumption that – independent of their cultural niches – infants share some fundamental expectations of social interactions, we put forth the assumption that a universal response exists for when a social interaction is interrupted. At the same time, we hypothesized that the size of the effect depends on the typicality of the interaction that precedes the adult partners’ interruption. To test these hypotheses, we conducted the Still-Face Paradigm (SFP) with infants (3- and 4.5-month-olds) from two cultural milieus, namely Münster (urban Germany) and the Kichwa ethnic group from the northern Andes region (rural Ecuador), as these contexts presumably offer different ways of construing the self that are associated with different parenting styles, namely distal and proximal parenting. Furthermore, we developed a paradigm that comes much closer to the average expected environment of Kichwa infants, the “No-Touch Paradigm” (NTP). Overall, the results support our initial hypothesis that the still-face effect is universal. Moreover, infants from both cultural milieus responded to the no-touch condition with a change in negative affect. At the same time, some of the infants’ responses were accentuated in a culture-specific way: Kichwa infants had a stronger response to an interruption of proximal interaction patterns during the NTP. While our findings underline infants’ universal predisposition for face-to-face interaction, they also suggest that cultural differences in internalized interactions do influence infant behavior and experience and, in turn, development.

## Introduction

From birth onwards, infants dynamically interact with their caregivers and develop as selves in relation to others. As the patterns of interaction between infant and caregiver are repeated in similar ways again and again, those interactions become internalized, raising implicit expectations about future actions, feelings, and sensations in infants (Stern, 2018). Independent of their cultural niches, infants are likely to share some fundamental expectations about social interaction. For example, across cultures infants experience that caregivers react contingently to their communicative signals to similar degrees (Kärtner et al., 2010), an experience that allows them to perceive themselves as causal social agents. At the same time, maternal contingent responses differ between cultures in their emphasis on distal (i.e., visual) and proximal (i.e., body touch and stimulation) modalities (Kärtner et al., 2010), which may lead to differences in the interactional routines that infants experience and internalize and the expectations that result from that social interaction.

The Still-Face Paradigm (SFP) designed by Tronick et al. (1978) is a standardized experimental setting in which a social partner, typically the mother, interrupts a specific format of social interaction, namely a distal face-to-face interaction with an infant, and becomes unresponsive. In most studies, coming predominantly from Western, Educated, Industrialized, Rich, and Democratic (WEIRD) cultures (Henrich et al., 2010), the partner’s still face evokes a so-called *still-face effect*, namely a decrease in gaze and positive affect and an increase in negative affect (Mesman et al., 2009). In accordance with the idea that contingent responsiveness is a key feature of early parenting (Fourment Sifuentes et al., 2021; Kärtner et al., 2010) and that “a sudden loss of responsiveness of the interaction partner touches upon deep-seeded characteristics of human interaction” (Mesman et al., 2009, p. 36), Mesman et al. (2009) propose that the still-face effect is universal.

In this context, we take the perspective of universality without uniformity (see also Shweder & Sullivan, 1993): We make the basic assumption that universally, infants respond to interrupted social interaction by decreases in gaze and positive affect and increases in negative affect. Furthermore, the magnitude of this effect should vary in culture-specific ways. Specifically, infants should react more strongly when the initial social interaction is closer to their everyday experience. To test these hypotheses, we complemented the SFP with a paradigm that reflects typical patterns of mother-infant interaction in other cultures, namely a proximal mode of social interaction.

### Parenting from a culture-sensitive viewpoint

Cross-cultural variability in parenting styles has been documented in a number of studies (Keller, 2007; LeVine, 1990). In accordance with Super and Harkness (1986), we regard customs of childcare such as carrying an infant on the back or mirroring emotions during arousal-eliciting face-to-face interactions as an elementary part of infants’ *developmental niche*, and, as such, customs of childcare are influenced by the other components of the niche, more precisely by parental ethnotheories and by physical and social settings as provided by caregivers. Moreover, parents’ socio-cultural orientations, such as their culture-specific *construals of the self*, play a major role in shaping their parenting styles during early infancy (Keller et al., 2004; Keller & Kärtner, 2013). According to Markus and Kitayama (1991), some people may have a more *independent self-construal* and others have a more *interdependent self-construal*. For those with an interdependent self-construal, the primary unit of consciousness lies in relationships, more specifically in the reciprocal interdependence between them and other actors, rather than in one’s inner self. By contrast, for persons with an independent self-construal, social situations still do affect the person’s attributes, traits, desires, and motives, but these things are the property of the individual, who is the primary unit of consciousness. With regard to intergenerational transmission paths, Markus and Kitayama (1991) suggest that “these construals of self are probably abstracted through early patterns of direct interactions with parents” (p. 246). In accordance with them, we conceptualize parenting styles as normative practices that foster the development of a specific sense of self.

Concerning infant experiences during everyday interactions and the internalization of those interactions (Stern, 2018), the question remains: Which aspects of parenting behavior are actually perceivable by the infant? Stern (2018) describes this “alphabet for sociocultural contextualization” (new introduction of the author, p. xxvii) as follows: “The repertoire comprises facial expressions, or the lack thereof; visual regards, or their avoidance; vocalizations, or silences; body orientations; physical distances; gestures; ways of being held; the rhythms, timing, and duration of acts and activities” (new introduction of the author, p. xxvii).

#### Parenting systems and cultural differences in parenting styles

In keeping with Stern’s analogy, different cultures have the same alphabet, but they use it to make different sentences. Similarly, addressing the first half year of an infant’s life, Keller and Kärtner (2013) introduced the component model of parenting, which differentiates six *parenting systems*, namely primary care (food, shelter, and hygiene), body contact (bodily proximity through holding and carrying), body stimulation (motor challenges through touch and movement), narrative envelope (use of language), face-to-face exchange (mutual eye contact), and object stimulation. According to the authors, those parenting systems are universal predispositions, but the probability of their (co-)occurrence varies with parents’ socio-cultural orientations, resulting in culture-specific *parenting styles*. Moreover, Keller and Kärtner (2013) identified four types of interactional mechanisms – attention, warmth, contingency, and responsiveness – that are also universal behavioral endowments. As in the case of cultural differences in modal patterns of contingent responses to infants’ communicative signals (Kärtner et al., 2010), these mechanisms manifest in culture-specific ways.

With regard to culture-specific parenting styles, infants from independent milieus frequently experience affect mirroring (Holodynski & Seeger, 2019) and the exchange of smiles during face-to-face interactions (Wörmann et al., 2014). By means of mirroring (emotional) cues during face-to-face interaction, caregivers promote psychological functions in infants (e.g., mentalization processes, self-realization, self**–**other differentiation; Fonagy, 2004; Kärtner, 2015) that play a fundamental role in this particular developmental niche (Keller & Greenfield, 2000; Keller & Kärtner, 2013; Stern, 2018; Super & Harkness, 1986) and form the basis for developing an independent self (Markus & Kitayama, 1991).

More research is needed about the development of self-realization and self–other differentiation in infants in interdependent cultural milieus who spend more time in bodily proximity and experience less face-to-face interaction (Keller & Kärtner, 2013). Infants who are attached to their mother’s body during large parts of the day, “come to expect that they will be watching others interact without receiving attention themselves” (LeVine, 1990), an interactional experience that might foster the development of selves-in-relation-to-others (Keller et al., 2004). In that sense, we propose that cross-cultural differences in parenting styles are functional for the development of a specific sense of self.

Overall, infants from cultures associated with an independent self-construal internalize a different repertoire of interaction experiences than infants from cultures associated with an interdependent self-construal. It follows that their implicit social expectations should also differ. Consequently, infants should react differently to different types of interactions and, especially, their interruption: The closer the interaction style is to their everyday experience, the more strongly they should react to the interruption thereof. More generally – based on fundamental processes of social interactions – we expect a universal response to the interruption of social interaction. However, the magnitude of infants’ responses should depend on the interactional routines that infants experience in their culture.

### First empirical evidence on cross-cultural differences in infants’ reactions during social interaction

Evidence for cross-cultural similarities and differences in infants’ attention and affect-expressive behavior comes from previous cross-cultural behavioral and observational studies on mother-infant interactions. For example, Kärtner et al. (2010) took a closer look at infants’ gazing at their mothers’ faces during postnatal weeks 4, 6, 8, 10, and 12: By contrasting educated urban middle-class families from Münster, Germany, with Nso families living in subsistence-based farming ecologies in rural Cameroon, the authors found that infants from the Münster sample looked at their mothers’ faces twice as long (when mothers themselves had established face-to-face contexts) than did infants from the Nso sample. Based on a re-analysis of the same samples, Wörmann et al. (2012, 2014) investigated the development of smiling and found a parallel increase in the duration of infant and maternal smiling during postnatal weeks 6 and 8 only in the Münster sample (see also LeVine, 1990). These results support the idea of culture-specific developmental paths that emerge during the second month of life on the grounds of early interactional experiences.

However, the question remains of whether the reported differences in infant behavior are due to differences in mothers’ interactive behavior (e.g., stimulation of affect) or whether those differences are developmental differences that would also manifest when adult stimulation is standardized. Therefore, cross-cultural similarities and differences in infants’ reactions during social interaction (and the interruption thereof) should also be addressed in standardized experimental settings.

### The still-face paradigm

Independent of their cultural niches, the sudden loss of contingent responsiveness, reciprocal exchange, and co-regulation of infant states should cause attentional and affective reactions in infants. Previous studies using the SFP typically focused on changes in gaze, positive affect, and negative affect as indicators for the still-face effect (Mesman et al., 2009).

After Tronick introduced the SFP in 1978 to the scientific community, it inspired a great amount of research. That is, researchers replicated the still-face effect in different samples, for example with infants between 1.5 months and 6 months of age (Bertin & Striano, 2006; Toda & Fogel, 1993) and in samples with risk factors such as maternal depression (Field et al., 2007) or prematurity (Segal et al., 1995). The paradigm was also applied with different interactive partners (mother, father, and experimenter) and with varying degrees of standardization.

Concerning the role of culture, most studies on the still-face effect are largely based on samples that are not representative of the world’s populations. To our knowledge, only six out of more than 85 studies employing the SFP (Mesman et al., 2009) have explored the still-face effect in non-WEIRD countries, including both urban and rural samples of mothers, fathers, and adult strangers and 6-week-old to 9-month-old infants from China, Taiwan, Japan, Ecuador, and Ghana (Handal et al., 2017; Hsu & Jeng, 2008; Kisilevsky et al., 1998; Li et al., 2019; Owusu-Ansah et al., 2019;Yato et al., 2008). The majority of these studies consistently report decreases in infants’ gaze and positive affect, at least for a subsample of infants (Owusu-Ansah et al., 2019), supporting the conclusion that infants across cultures react to the interruption of social interaction in similar, potentially universal ways.

However, in most studies, it remains unclear to what exactly the infants responded to, because the intensity and modal patterns of stimulation (per instruction or as shown by interactive partners) were not further specified: More specifically, it is unclear whether infant responses represented a *pure* still-face effect or whether they were instead responding to the interruption of more proximal modes of interaction such as the loss of tactile contact. With the exception of Handal et al. (2017), adult partners were instructed to engage in *normal* interaction (framed as face-to-face interactions) with infants before interrupting social interaction. Overall, it seems that authors did not standardize the interactive behavior of the adult partner during the baseline of the SFP. Therefore, it remains unclear to what extent infants react differentially – that is, in culture-specific ways – to different modal patterns of interaction that are more or less characteristic of their everyday experience.

For the purpose of the present cross-cultural study, we therefore chose experimenters (native female strangers) to be interactive partners and standardized their interactive behavior: During the baseline of the SFP, they exclusively used distal modes of interaction, which are frequently experienced by infants from independent cultural milieus. Moreover, we developed a second paradigm that comes much closer to the average expectable environment (LeVine, 1990) of infants from interdependent milieus, the No-Touch Paradigm (NTP): During the baseline phase, the experimenter touched and held the infant and then interrupted tactile contact (no-touch phase). We conducted the SFP and the NTP longitudinally when infants were 3 and 4.5 months old, an age range during which the still-face effect has consistently been found (Mesman et al., 2009). Infants and their families came from two cultural milieus with presumably different construals of the self (Markus & Kitayama, 1991); in particular, we recruited families with high degrees of formal education living in Münster (urban Germany) and families who identify themselves as Kichwas and who live in communities around the neighboring cities of Otavalo (approx. 31,000 inhabitants) and Cotacachi (approx. 7,500 inhabitants; rural Ecuador).^1^ Below, we take a closer look at these two different cultural milieus before we finally outline our hypotheses.

### Parenting in urban Germany and rural Ecuador

Münster is a middle-sized city with approximately 311,000 inhabitants in North-Western Germany (City of Münster - Department of Urban Planning, 2019). With its 65,000 students (City of Münster - Department of Urban Planning, 2019), Münster is considered a city of science and is known for its high quality of life (Prognos AG, 2018). Germany is an immigration country: The proportion of people with a migration background in Münster amounts 23.22% (Stadt Münster - Stadtplanungsamt, 2020). In accordance with previous studies (Wörmann et al., 2012, 2014) and with Markus and Kitayama (1991), who attributed the independent view of the self to a large proportion of American and European cultures, we expect families in Münster to emphasize the developmental goal of independence. For instance, this becomes explicit in socialization goals such as “develop personal interests and talents” and “express [your] own preferences very clearly,” which were rated as being more important for child development by mothers from urban Germany than by rural Indian mothers and mothers living in rural Cameroon (Kärtner et al., 2012). Caregivers in Münster and other highly educated German urban middle-class samples typically use distal parenting strategies with a focus on object stimulation and face-to-face interaction (Keller et al., 2004; Keller & Kärtner, 2013).

About 60,000 people who identify themselves as Kichwas live in *comunidades* in the cantons of Otavalo and Cotacachi, which are located in the northern Andes of Ecuador at a height of 2,500–3,000 m (Lattrich, 2006). Agriculture and ethnic handicrafts are the main economic sources of income for the Kichwas, whereas many handicrafts are sold at the famous market in Otavalo to international tourists or sold by Kichwas who travel around the world (Lattrich, 2006). Otavalo and Cotacachi both pride themselves as the heart and center of the indigenous intellectuals in Ecuador and the Kichwas in both cantons are characterized by an ethnic identity that is connoted very positively (Lalander, 2010; Lattrich, 2006).

As proposed by Markus and Kitayama (1991), the interdependent view of the self is characteristic of many Asian as well as Latin American cultures. This view closely resembles the concept of reciprocity, a cultural value that lays the foundation for communal life in Indigenous communities across the Andean region, like in the communities where we conducted our study (De la Torre Amaguaña & Sandoval Peralta, 2004; Tousignant & Maldonado, 1989). Despite ongoing cultural changes that are brought about by foreign factors associated with globalization, the value of reciprocity has not lost importance but might indeed manifest itself differently. As Lattrich (2006) pointed out, those foreign factors are adapted to cultural circumstances and values in the moment of their acquisition. An example of this creative and dynamic transformation process are new weaving workshops in Otavalo, where prosperous Otavalos employ family members, neighbors, and friends from the same ethnic group, nominally forming part of the social network and of reciprocal relationships (Lattrich, 2006).

In comparison to infants from highly educated German urban middle-class samples, Kichwa infants spend more time in body contact with the parent. For example, many Kichwa mothers wrap their infants in a blanket and attach them to their back for substantial parts of the day, a custom inherited from earlier generations. According to Yuri Amaya Guandinango (Kichwa woman, living in Cotacachi, Ecuador; personal communication, 28 March 2021) this custom is associated with the protection from *negative energies* (ancestral knowledge) and with new mothers’ traditional re-engagement in labor activities after 45 days of rest (see also Lancy, 2011). The custom to attach the baby to the mother’s back, her chest, or her hip, for example with a *manta* *pouch*, has been reported for various Indigenous groups in the Andes (Lancy, 2011).

Based on these findings, we assumed that 3- and 4.5-month-old infants from Münster and the Kichwa ethnic group would differ in their average expectable environments. Moreover, the interactional experiences that infants have in their developmental niches should influence their attentional and affective reactions during the SFP and the NTP. More precisely, the size of the still-face effect and the no-touch effect should depend on the typicality of the interaction that precedes the adult partners’ interruption.

To summarize our hypotheses, we expected, first, that 3- and 4.5-month-old infants from Münster and the Kichwa ethnic group would display the still-face effect as indicated by a decrease in social gaze and positive affect and an increase in negative affect from the baseline to the still-face phase. Second, we hypothesized that infants would show similar response patterns when confronted with the sudden interruption of touch. That is, we expected infants from both cultural milieus and at both ages to display a *no-touch effect*. Third, we expected culture-specific patterns with respect to the size of both effects: We expected infants from Münster to respond more strongly to the interruption of distal parenting in comparison to Kichwa infants. Likewise, we expected Kichwa infants to display a stronger response to the interruption of proximal parenting as compared to infants from Münster.

## Methods

### General procedures

The present study was part of a larger cross-cultural longitudinal project on early mother-infant interaction, and data assessment took place from 2017 to 2018. In Ecuador, we conducted the study in cooperation with the University of Otavalo and the Union of Farmer and Indigenous Organizations of Cotacachi (UNORCAC). The project was approved by the scientific commission of the University of Otavalo. Research processes such as timing of the first data assessment, arrangement of contact to the families, and sharing of the research findings with the community were subject to discussion with the Ecuadorian research team during the pilot phase and were adapted to local customs. For the main data collection of the present study, we selected mothers and infants who identified themselves as Kichwas, who lived in communities in the larger surroundings of Cotacachi or Otavalo, and who gave birth no more than 6 weeks prior to the start of the study. The hospitals of Cotacachi and Otavalo provided us with information about newborn children. A local research assistant visited the families, informed them about the study, and invited the mothers to participate. In the overall project, families received food provisions (sugar, rice) during every visit, a monetary refund as a compensation for their loss of income, and a collection of the videotaped mother-child interactions at the end of the data assessment.

In Germany, we included mothers (and infants) who lived in the city of Münster and who recently gave birth. Mothers who had migrated to Münster from abroad were also included. We contacted families by post after receiving their contact information from the local registration office or invited them personally during prenatal classes. Families in Münster were rewarded for their collaboration in the overall project with a collection of their videotaped interactions and a little present for the infant.

### Participants

Because we were interested in meaningful effects (i.e., medium to large effect sizes with *f* ^2^ > .30), sample size was calculated based on a corresponding power analysis with GPower for each mixed analysis of variance (i.e., test family: F tests; statistical test: ANOVA, repeated measures, within-between interaction), based on the following input parameters: two tails, *f* ^2^ = .30, α = .05, power (= 1 - β) = .80, number of groups = 2, number of measurements = 2, correlation among repeated measures = .5, nonsphericity correction ε = 1. The result indicated that a total sample size of *N* = 24 would be sufficient to detect corresponding effects. Regarding the samples from urban Germany (SFP and NTP at 3 and 4.5 months), we did not achieve the necessary sample sizes, because the drop-out rates were higher than anticipated.

A total of 28 families from urban Germany and 31 families from rural Ecuador participated in at least one of the four assessments (SFP and NTP at 3- and 4.5-month-olds). Given the focus of our research question, we included all assessments in which infants successfully completed the baseline and the still-face/no-touch phase. From the 118 potential sessions with two assessments each (NTP and SFP, fixed order), one session at 3 months from Ecuador and one session at 4.5 months from Germany did not take place, either because of regulatory difficulties associated with the infant (excessive crying, *n* = 1) or because a family moved in with a family member for several weeks (*n* = 1).

During the 116 data collection sessions that did occur, further assessments were not completed or had to be excluded for different reasons: (i) infants were fussy or cried before the assessment and were not ready to be separated from the parent and interact with a stranger (*n* = 7 at 3 months and *n* = 3 at 4.5 months), (ii) assessments were interrupted because infants cried consecutively for more than 10 s (*n* = 5 at 3 months and *n* = 5 at 4.5 months), (iii) assessments were excluded post hoc because the experimenter did not strictly follow the protocol (e.g., length of baseline less than 60 s (instead of 90 s) long; *n* = 10 at 3 months and *n* = 8 at 4.5 months), (iii) error in data storage (*n* = 2 at 3 months), (iv) whine/whimper for at least 20% of the baseline time (*n* = 2 at 3 months and *n* = 2 at 4.5 months).

This resulted in a final set of *N* = 91 still-face assessments (3 months: *n*_MS_ = 20, *n*_KI_ = 24; 4.5 months: *n*_MS_ = 20, *n*_KI_ = 27) and *N* = 90 no-touch assessments (3 months: *n*_MS_ = 20, *n*_KI_ = 26; 4.5 months: *n*_MS_ = 18, *n*_KI_ = 26).

### Demographics and description of cultural milieus

Seven weeks after the birth of their child, mothers were interviewed by local research assistants regarding the families’ demographic contexts, time budgets and daily routines. Demographic information about the two samples is presented in more detail in Table 1.

**Table 1:** Demographic information and description of cultural milieus

Sociodemographic variable		Cultural milieu		Statistical significance
		Münster % or *M* (*SD*)	Kichwa% or *M* (*SD*)	
Gender (% girls)		39.3%	63.3%	*χ*^*2*^ = 3.35, *ɸ* = -.24
Parity (% firstborn)		57.1%	33.3%	*χ*^*2*^ = 3.32, *ɸ* = .24
Age mothers (y)		33.39 (3.66)	28.53 (7.30)	*t* = -3.24^**^, *d* = 0.83
Age fathers (y)		36.54 (5.77)	32.43 (8.18)	*t* = -2.10^*^, *d* = 0.64
Migratory experience mothers		*N* = 5	*N* = 0	*t* = -2.87^*^, *p* = .008
Time since mothers migrated (y)		*Mdn* = 25.00 *MIN* = 4.00 *MAX* = 32.00	*-*	
Partnership status	Married	71.4%	70.0%	
	Living with partner	28.6%	16.7%	
	Single partners	0.0%	13.3%	
Household sizes		3.57 (0.88)	7.33 (2.82)	*t* = 6.95^*^, *d* = 1.83
Number of siblings		0.57 (0.88)	1.60 (1.99)	*t* = 2.57^*^, *d* = 0.66
Formal education, mothers (y)		15.50 (2.85)	8.70 (3.97)	*t* = -7.45^*^, *d* = 1.96
Formal education, fathers (y)		15.75 (3.43)	7.54 (4.20)	*t* = -7.90^**^, *d* = 2.13
Acquisition of a profession,^a^ mothers		Yes: 100% No: -	Yes: 60% No: 40%	*χ* ^*2*^= 14.12^**^, *ɸ* = .49

^a^Professions including formally acquired and self-learned skills

Samples did not differ significantly with regard to parity or infant gender. From the final sample, 39.3% of the Münster sample and 63.3% of the Kichwa sample were girls. In the Münster sample, there were descriptively more firstborn children (57.1%) than in the Kichwa sample (33.3%, see also Table 1). Mothers and fathers from Münster were significantly older than Kichwa parents. Five mothers living in Münster had migrated from abroad – three as children and two as adults – from Kazakhstan, Sri Lanka, Russia, Upper Silesia, or Hungary, whereas none of the Kichwa mothers had (see Table 1 for more details). The majority of mothers in both samples were married. Household sizes were significantly larger in the Kichwa sample compared to the Münster sample, and Kichwa infants had significantly more siblings than infants from Münster. Parents’ years of formal education differed significantly across cultures: Mothers and fathers in Münster received more years of formal education than Kichwa mothers and fathers. Of the mothers from rural Ecuador, 60% had acquired a profession (including formally acquired and self-learned skills), while 100% of the participants from Münster had acquired a profession. Typical professions amongst Kichwa mothers were agriculture (16.7%), fabrication of ethnic handicrafts (13.3%), and textile manufacture (13.3%). For mothers from Münster, the most frequent professions were business administrators (14.3%), teachers (10.7%), workers in healthcare (e.g., physiotherapist, 10.7%), academics in the humanities (10.7%), and psychologists (10.7%). We also asked mothers where their youngest child stays while they engage in their daily routines, and cultures differed significantly with regard to the location that mothers named most frequently, *χ*^*2*^(2) = 12.35, *p* = .002, ɸ = .46: In the Münster sample, 78.6% percent of infants were mainly located at a bodily distance from the mother (e.g., lying in front/next to the mother, in baby carriage), 7.1% were mainly in bodily proximity (e.g., carrying, holding), and 14.3% experienced both positions equally. The locations of Kichwa infants were equally distributed, with 33.3% of infants experiencing bodily proximity and bodily distance equally, 33.3% more frequently in bodily proximity (e.g., while the mother is taking care of the animals), and 33.3% more frequently located at a bodily distance from the mother.

### No-touch and still-face assessments

The participating families were informed about the data protection policy and signed an informed consent before the first data assessment. Two local research assistants visited the families at their homes, when infants were 3 and 4.5 months old. The modified still-face procedures (SFP and NTP) were realized in one session. The experimenter, a female adult stranger, began with the NTP, and – after a pause during which the infant could interact with the parent – continued with the SFP. The stranger was the same person at 3 and 4.5 months. At the beginning of both assessments, infants sat in a child seat and the experimenter sat in front of the seat with no toys, facing her/him. A GoPro Hero camera was installed on the backrest of the child seat, filming the experimenter, and a Panasonic HC Camcorder camera was placed on a tripod, filming the infant. The second research assistant gave a signal when the next phase of the experiment started.

In both paradigms, there was a baseline phase for 120 s, in which the experimenter interacted with the infant proximally (NTP) or face-to-face (SFP). During baseline, the intensity of stimulation by the experimenter was moderate during the first 60 s (baseline 1) and was then increased for the remaining 60 s (baseline 2). The experimenter then suddenly stopped interaction (interruption phase) and remained unresponsive for 90 s. During the reunion, the experimenter resumed social interaction as in the baseline for 120 s.

#### No-touch paradigm

During baseline 1, the experimenter touched the feet, the arms/hands and/or the upper body of the infant, contingently encouraging and supporting his/her movements. During baseline 2, the experimenter lifted the infant from the infant seat and held him/her in her arms, thereby maximizing body contact. During the interruption phase, the experimenter put the infant back in the infant seat and remained unresponsive (no eye contact, neutral face).

#### Still-face paradigm

During the baseline, the experimenter engaged in reciprocal face-to-face interaction, contingently mirroring his/her facial expressions and encouraging infant smiles. The intensity of smiles of the experimenter increased from baseline 1 to baseline 2. During the interruption phase, the experimenter remained unresponsive, with a neutral face, while keeping eye contact with the infant (no body contact).

To clearly separate proximal from distal behavior, we instructed the experimenter to keep a neutral facial expression and to look past the infant (focusing on a spot behind the infant) during all phases of the NTP and to refrain from body contact during all phases of the SFP. In both paradigms, the use of language was adapted to the degree of stimulation, and the experimenter remained quiet during the no-touch and still-face phase.

#### Behavioral coding and reliabilities

Based on an interval coding approach with 1-s intervals, infant gaze and affective reactions (as expressed by vocalizations and facial expressions) were coded during the baseline and the interruption phase of the SFP and the NTP, using Mangold Interact (version 16.1.5.8). A first inspection of the data showed that facial expressions and gaze could not be coded for large parts of baseline 2 of the NTP, because the infants’ faces were covered by the body of the experimenter while she was holding them. As a consequence, we decided to exclude baseline 2 from the NTP coding and analyses. The average lengths of the phases were as follows: baseline of the NTP = 61 s, baseline of the SFP = 122 s; the no-touch phase = 86 s, and the still-face phase = 89 s.

##### Gaze

We used the following categories to code infant attentive reactions: (1) gazing at the experimenter (gaze is focused on her face during the complete 1-s interval), (2) not gazing at the experimenter (not focused on her face, eyes opened), (3) switching gaze (between focusing on the experimenter’s face and not focusing), (4) eyes closed, and (5) gazing could not be coded (e.g., due to hidden face).

##### Vocalizations

For each 1-s interval, one of the following codes was given: (1) no vocalization, (2) neutral vocalization (containing neither a positive nor a negative valence), (3) negative vocalization (whining, whimpering, crying, but also angry and sad vocalizations), (4) positive vocalization (cooing, low intensity sounds of contentment, mild laughter, sounds of contentment with very positive valence and open laughter), (5) vocalization could not be coded (e.g., not audible due to background noise), and (6) *other* sounds (e.g., unvoiced sounds, vegetative sounds such as hiccups). In the case of changes in the valence of vocalizations during the same 1-s interval, negative vocalizations were prioritized over positive vocalizations, and positive vocalizations were prioritized over neutral vocalizations.

##### Facial expressions

We used the following categories to code infant facial expressions: (1) neutral facial expression, (2) negative affect (aversive responses: brow knitting, lower lip raising, horizontal stretching of lip corners), (3) positive affect (smiles as indicated by varying degrees of raising or sideway movements of lip corners, eye constriction, mouth opening and raising of cheeks), (4) face covered (by the body of the experimenter, while she is holding him/her), and (5) facial expression could not be coded (impossible to identify due to insufficient light, face being hidden, etc.). In the case of changes in the valence of facial expressions during the same 1-s interval, negative affect was prioritized over positive affect, and positive affect was prioritized over neutral facial expressions.

Reliabilities. We calculated interrater reliabilities between a gold standard and two independent coders for 16% of the 181 videotaped assessments resulting in approximately 4,190 independently coded 1-s intervals. Within this set, cultural milieus, experimental procedures (SFP/NTP) and measurement points were equally distributed. For gaze, vocalizations and facial expressions, Cohen’s kappas were computed after excluding non-codable intervals and all κs exceeded .64, with 4 out of 5 κs ≥ .73, indicating a substantial strength of agreement (Landis & Koch, 1977).

#### Dependent variables for infant gaze and affect

For the analyses, we computed relative frequencies of 1-s intervals from all codable 1-s intervals per phase (i.e., baseline (baseline 1 for the NTP and baseline 1+2 for the SFP) and interruption phase) and task. *Infant gaze* was computed as the relative frequency of the code gaze at the experimenter (i.e., number of intervals with infant gaze divided by all codable intervals), *positive affect* was defined as the relative frequency of intervals in which positive vocalization or positive facial expression was coded, and *negative affect* was defined as the relative frequency of intervals in which negative vocalization or a negative facial expression was coded.

### Plan of analysis

We used SPSS (version 26) for the analysis of infants’ reactions during the SFP and the NTP. We conducted mixed analyses of variance (i.e., mixed ANOVAs) to test the decrease of positive affect and the increase in negative affect and gaze (dependent variables) with phase (baseline vs. interruption) as a within-subject factor; infants’ cultural milieu (Münster vs. Kichwa) was the between-subject factor. In case of significant interactions, we conducted post hoc *t*-tests.

## Results

Due to the pattern of missing data (i.e., single assessment missing for one of the tasks at one of the two ages), we computed separate ANOVAs for each measurement point (age: 3 and 4.5 months) and for each task (SFP and NTP). Prior to data analysis, we used box plots to detect univariate outliers and transformed extreme outliers (three times the interquartile range) by assigning them a value that was .01 larger than the highest score within the distribution (not being an outlier yet; (Tabachnick & Fidell, 2007)). In the following, we report the results of the ANOVAs based on untransformed values, because analyses of transformed and untransformed values led to identical patterns of results with one exception (see also Footnote 2). Preliminary analyses had shown that neither main effects of gender and parity nor their interactions with culture and phase were significant when including them as an additional factor into separate ANOVAs, with only few exceptions (see Appendix 1). Therefore, we decided to drop them from the final analyses. Finally, the pattern of results was identical when excluding mothers who had migrated to Germany (see Appendix 2 for details).

### Still-face effect at 3 and 4.5 months

**Fig. 1:**
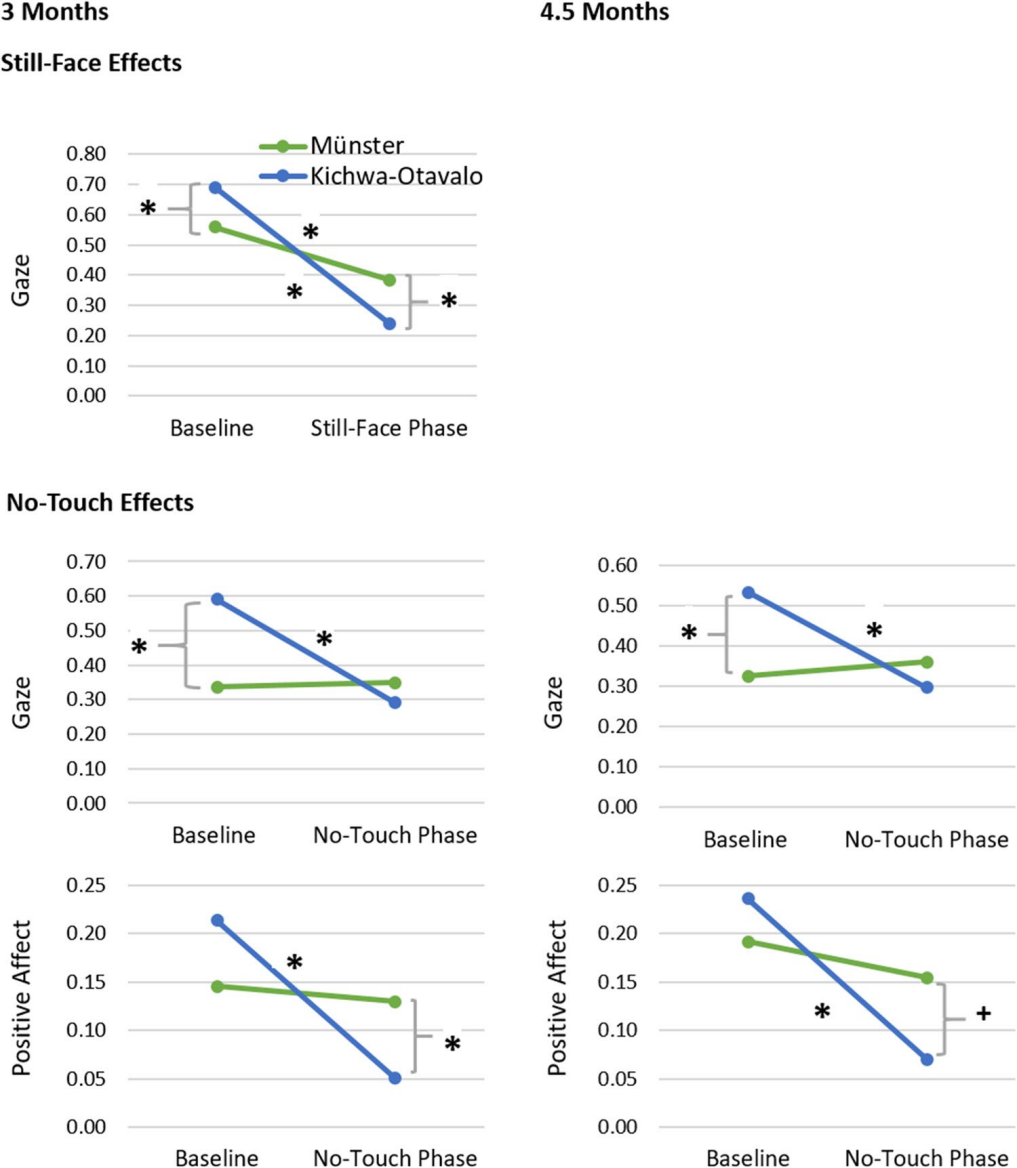
Visualizing significant cultural milieu × phase interactions for the still-face and the no-touch effects. *Note.* Relative frequencies of infant gaze at the experimenter and positive affect during the still-face experiment and the no-touch experiment at 3 and 4.5 months: Interaction of cultural milieu × phase according to post hoc *t-tests*. Scales ranging between 0 and 1 (= 100% of codable intervals)

Regarding infant gaze at 3 months of age, there was a significant effect of phase, *F*(1, 42) = 86.97, *p* < .001, *η*^*2*^ = .67, but no effect of cultural milieu, *F*(1, 42) = 0.02, *p* =.879, *η*^*2*^ = .00. The effect of phase was further qualified by a significant cultural milieu × phase interaction, *F*(1, 42) = 16.36, *p* < .001, *η*^*2*^ = .28, indicating that, contrary to our hypotheses, the still-face effect was more pronounced in the Kichwa sample (see Fig. 1). Post hoc *t*-tests yielded a significant decrease of infant gaze in both the Münster, *t*(19) = 4.46, *p* < .001, *d* = 1.00, and the Kichwa sample, *t*(23) = 8.71, *p* < .001, *d* = 1.78. Comparing the two cultures directly, post hoc *t*-tests indicated that Kichwa infants gazed longer at the experimenter during the baseline than infants from Münster did, *t*(42) = 1.73, *p* =.091, *d* = 0.52, but significantly shorter during the still-face phase, *t*(42) = -2.19, *p* =.033, *d* = 0.67.

Regarding positive affect at 3 months of age, the ANOVA yielded a significant main effect of phase, *F*(1, 42) = 43.62, *p* < .001, *η*^*2*^ = .51, based on a decrease in positive affect from baseline to the interruption phase across samples. The main effect of cultural milieu was not significant, *F*(1, 42) = 0.80, *p* = .375, *η*^*2*^ = .02, and the interaction of cultural milieu × phase, *F*(1,42) = 0.07, *p* = .791, *η*^2^ = .00, was not significant either.

Concerning negative affect at 3 months of age, the main effect of phase was marginally significant, *F*(1, 42) = 3.89, *p* = .055, *η*^*2*^ = .09, pointing towards an increase in negative affect from baseline to the interruption phase across samples.^2^ The ANOVA yielded no significant main effect of cultural milieu, *F*(1,42) = 0.07, *p* = .798, *η*^2^ = .00, nor a significant interaction of cultural milieu × phase, *F*(1,42) = 0.00, *p* = .990, *η*^2^ = .00.

At 4.5 months of age, there were – as hypothesized – significant effects of phase on all scores, indicating significant decreases in infant gaze, *F*(1, 45) = 57.86, *p* < .001, *η*^*2*^ = .56, and positive affect, *F*(1,45) = 26.55, *p* < .001, *η*^2^ = .37, and an increase of negative affect, *F*(1,45) = 16.26, *p* < .001, *η*^2^ = .27. Furthermore, there were no significant effects of cultural milieu, *F*s(1,45) < 0.56, *p*s > .459, *η*^2^s < .02, or significant cultural milieu × phase interactions, *F*s(1,45) < 2.48, *p*s > .122, *η*^2^s < .06.

Overall, and in support of our first hypothesis, these findings showed significant changes for infant gaze and both positive and negative affect during the still-face phase at both ages and in both cultural milieus. At 3 months of age, the change in gaze was, contrary to our second hypothesis, more pronounced in the Kichwa infants. Means and standard deviations for attentional and affective reactions during the SFP at 3 and 4.5 months are reported in Table 2.

**Table 2:** Means and standard deviations for attentional and affective reactions during the still-face paradigm at 3 and 4.5 months

	Baseline		Interruption phase		Partial η^2^		
	Münster	Kichwa	Münster	Kichwa	CM	P	CM × P
3 months	*M* (*SD*)	*M* (*SD*)	*M* (*SD*)	*M* (*SD*)			
Gaze [0-1]	.56 (.24)	.69 (.24)	.38 (.22)	.24 (.22)	.00	.67**	.28**
Positive affect [0-1]	.29 (.19)	.25 (.16)	.11 (.11)	.08 (.11)	.02	.51**	.00
Negative affect [0-1]	.08 (.13)	.07 (.11)	.14 (.28)	.13 (.19)	.00	.09	.00
4.5 months							
Gaze [0-1]	.45 (.29)	.50 (.28)	.26 (.20)	.21 (.18)	.00	.56**	.05
Positive affect [0-1]	.22 (.16)	.21 (.19)	.10 (.15)	.06 (.09)	.01	.37**	.01
Negative affect [0-1]	.02 (.04)	.03 (.05)	.14 (.18)	.11 (.16)	.00	.27**	.01

*Note*. Still-face effect at 3 months: *N* = 20 for the Münster sample and *N* = 24 for the Kichwa sample.

At 4.5 months: *N* = 20 for the Münster sample and *N* = 27 for the Kichwa sample.

* *p* < .05; ** *p* < .01

### No-touch effect at 3 and 4.5 months

Since the pattern of results for the no-touch effect was identical for both age groups, the following findings will be reported – different from the structure above – in a summarized fashion across ages, separate for each infant behavior.

Regarding infant gaze, there was a significant effect of phase at 3 months of age, *F*(1, 44) = 9.83, *p* = .003, *η*^*2*^ = .18, and at 4.5 months of age, *F*(1, 42) = 4.95, *p* = .032, η^2^ = .11. However, there was no effect of cultural milieu at both ages, *F*s < 1.74, *p*s > .191, *η*^2^s < .05. The effect of phase was further qualified by a significant cultural milieu × phase interaction at 3 months of age, *F*(1, 44) = 11.60, *p* = .001, *η*^*2*^ = .21, and at 4.5 months of age, *F*(1, 42) = 9.13, *p* = .004, *η*^*2*^ = .18, indicating that the no-touch effect for gaze was found in the Kichwa sample but not in the Münster sample (see Fig. 1 and Table 3). Post hoc *t*-tests yielded a significant decrease in infant gaze in the Kichwa sample at 3 months, *t*(25) = 5.61, *p* < .001, *d* = 1.10, and at 4.5 months, *t*(25) = 4.57, *p* < .001, *d* = 0.90, whereas gaze did not decrease in the Münster sample at both ages, *t*(17) and *t*(19) < -0.46, *p*s > .652, *d*s < 0.11. Comparing the two cultures directly, post hoc *t*-tests indicated that Kichwa infants gazed longer at the experimenter during the baseline at 3 months, *t*(44) = 2.84, *p* =.007, *d* = 0.84, and at 4.5 months, *t*(42) = 2.42, *p* =.020, *d* = 0.74. At both ages, samples did not differ with regard to infant gaze at the experimenter during the no-touch phase, *t*s < -0.87, *p*s >. 390, *d*s < 0.27.

**Table 3:** Means and standard deviations for attentional and affective reactions during the no-touch paradigm at 3 and 4.5 months

	Baseline		Interruption phase		Partial η^2^		
	Münster	Kichwa	Münster	Kichwa	CM	P	CM × P
3 months	*M* (*SD*)	*M* (*SD*)	*M* (*SD*)	*M* (*SD*)			
Gaze [0-1]	.34 (.28)	.59 (.31)	.35 (.27)	.29 (.28)	.04	.18**	.21**
Positive affect [0-1]	.15 (.17)	.21 (.21)	.13 (.17)	.05 (.09)	.00	.16**	.12*
Negative affect [0-1]	.05 (.10)	.06 (.10)	.21 (.27)	.22 (.28)	.00	.24**	.00
4.5 months							
Gaze [0-1]	.32 (.28)	.53 (.28)	.36 (.26)	.30 (.22)	.03	.11*	.18**
Positive affect [0-1]	.19 (.26)	.24 (.22)	.16 (.20)	.07 (.10)	.00	.26**	.13*
Negative affect [0-1]	.03 (.06)	.01 (.03)	.17 (.26)	.16 (.21)	.00	.31**	.00

No-touch effect at 3 months: *N* = 20 for the Münster sample and *N* = 26 for the Kichwa sample. At 4.5 months: *N* = 18 for the Münster sample and *N* = 26 for the Kichwa sample

* *p* < .05; ** *p* < .01

With respect to positive affect, there was a significant effect of phase at 3 months of age, *F*(1,44) = 8.39, *p* = .006, *η*^2^ = .16, and at 4.5 months of age, *F*(1, 42) = 14.54, *p* < .001, *η*^*2*^ = .26. However, there was no effect of cultural milieu at both ages, *F*s < 0.19, *p*s > .674, *η*^2^s <.01. The effect of phase was further qualified by a significant cultural milieu × phase interaction at 3 months of age, *F*(1, 44) = 5.71, *p* = .021, *η*^*2*^ = .12, and at 4.5 months of age, *F*(1, 42) = 6.06, *p* = .018, *η*^*2*^ = .13, indicating that the no-touch effect for positive affect was found in the Kichwa sample but not in the Münster sample (see Fig. 1 and Table 3). Post hoc *t*-tests yielded a significant decrease of positive affect in the Kichwa sample at 3 months, *t*(25) = 4.57, *p* < .001, *d* = 0.90, and at 4.5 months, *t*(25) = 4.59, *p* < .001, *d* = 0.90. Positive affect did not decrease in the Münster sample at both ages, *t*s < 0.99, *p*s > .336, *d*s < 0.24. Comparing the two cultures directly, post hoc *t*-tests indicated that infants from Münster showed more positive affect during the no-touch phase at 3 months, *t*(44) = - 2.11, *p* =.040, *d* = 0.63, and showed the same tendency at 4.5 months, *t*(23.08) = -1.76, *p* =.091, *d* = 0.54. At both ages, samples did not differ with regard to positive affect during the baseline, *t*s < 1.21, *p*s > .232, *d*s < 0.36.

Concerning infant negative affect, the main effect of phase was significant at 3 months of age, *F*(1, 44) = 14.23, *p* < .001, η^2^ = .24, and at 4.5 months of age, *F*(1, 42) = 18.86, *p* < .001, *η*^*2*^ = .31. There was no effect of cultural milieu at both ages, *F*s < 0.08, *p*s > .789, *η*^2^s < .01, nor a significant interaction of cultural milieu × phase, *F*s < 0.02, *p*s > .895, *η*^2^s = .00.

Overall, these findings give partial support to our second hypothesis that there is a *no-touch effect* at both ages and in both cultural milieus: While there was a clear no-touch effect across ages and behavioral indicators (i.e., infant gaze and both positive and negative affect) in the Kichwa sample, this effect was less pronounced in Münster. More specifically, Münster infants only showed significant increases in negative affect at both ages. This differential pattern, in turn, was in line with the third hypothesis, namely that Kichwa infants display a stronger response to the interruption of proximal parenting as compared to infants from Münster.

## Discussion

In the present cross-cultural study, we tested infants’ reactions to an unresponsive partner. In support of a “universality without uniformity” perspective, we found that infants from both cultural milieus responded to the still-face and the no-touch phase with a change in at least infant gaze or affect. At the same time, there were culture-specific accentuations of infants’ responses; in particular, Kichwa infants responded more strongly to an interruption of proximal interaction patterns than did infants in Münster. In the following, we discuss the main findings in turn.

### Still-face effect

In the SFP, we found significant changes in infant gaze and affect at both ages and in both cultural milieus. Those findings serve as further evidence for the robustness of the still-face effect across cultures, also including the expected increase in negative affect (Hsu & Jeng, 2008; Yato et al., 2008). Contrary to our second hypothesis on the still-face effect, we found no evidence for a stronger effect in the Münster sample. While there were similar changes for both affect and gaze in the two cultural milieus, the change in gaze was even more pronounced in Kichwa infants at 3 months of age.

More specifically, the stronger decrease in gaze from baseline to the interruption phase at 3 months of age was composed of two effects, first of Kichwa infants gazing longer at the experimenter’s face during the baseline and, second, their gazing at the experimenter’s face for a shorter time during the still-face phase than the Münster infants. Taking those findings into account, one interpretation is that during the baseline, the attention of Kichwa infants was captured more strongly by the experimenter’s face-to-face interaction – mirroring infants’ facial expressions and encouraging infant smiles – because the amount of stimulation exceeded their everyday experiences, leading to heightened interest. Once the experimenter interrupted this high-intensity face-to-face way of interacting, these infants were gazing at the experimenter significantly less than Münster infants, because – based on their interactional routines with caregivers – they were less routinized in seeking and establishing mutual gaze than infants from Münster and, as a consequence, returned to their habitual gaze pattern that, in interdependent cultures, is less focused on others’ faces (see, e.g., Kärtner et al., 2010).

Since infants’ responses were not more pronounced in the Münster sample, one could argue that the pattern found, namely significant changes for all indicators across ages and cultures, indicates nothing more than a universal sensitivity for human faces and an interest in face-to-face interaction, especially during the second and third months of life (Lavelli & Fogel, 2002, 2005), that is independent of infants’ interactional history. While we agree that there certainly is a universal predisposition for face-to-face interaction, we would argue that, at the same time, the culture-specific gaze pattern of 3-month-old Kichwa infants – indicating a strong interest in face-to-face interaction and a return to habitual gaze patterns after the face-to-face interaction ends – suggests that cultural differences in internalized interactions do have implications for infant behavior and experience and, in turn, development.

Overall, these findings provide clear support for a universal still-face effect with very similar infant responses in both cultural milieus, namely a Western urban middle-class sample and an Indigenous-heritage Kichwa sample from a rural Andes region.

### No-touch effect

When interacting with infants in a more proximal mode during the baseline, namely by contingent touching and holding with synchronized vocalizations, as realized in the no-touch paradigm, an interruption leads to the pattern of responses that we expected from a “universality without the uniformity” perspective: We again see a universal response in the sense that at both ages and in both cultural milieus, there was a significant change in at least one aspect of infant gaze or affect. At the same time, the response was more pronounced in Kichwa infants, the cultural milieu that emphasizes proximal interaction styles during everyday routines.

The similar increases in negative affect across phases suggest that touch and holding with synchronized voice during the baseline of the NTP conveys (emotional) contact to infants, and possibly togetherness and warmth (Keller et al., 2004), and that the sudden loss thereof caused feelings of insecurity in infants from both cultural milieus. The importance of the sense of touch during early infancy and across cultures is in line with H. Papoušek and Papoušek’s (1987) concept of intuitive parenting and Keller and Kärtner’s (2013) component model of parenting (see also Bigelow & Williams, 2020). Similarly, Stack and Muir (1990) modified the traditional SFP by introducing the use of touch during the still-face phase: They reported a significant reduction of the still-face effect in this condition (as compared to the standard still-face, no-touch condition), which supports the interpretation that – by means of maternal touch – infants kept *contact* with her, even when her face remained unresponsive. Apart from the increase in negativity, infants from the two samples responded differently to the interactional offer: The Münster infants’ gaze at the experimenter and positive affect did not change from the touch to the no-touch phase. Together with the finding that infant smiling and gaze was relatively low in the baseline of the NTP, this indicates that infants from Münster might not have recognized the experimenter’s behavior (i.e., encouraging and supporting infants’ movements while keeping a neutral facial expression and looking past the infant plus establishing close body contact) as a reciprocal social interaction, because the crucial cue – mutual eye contact (Lavelli & Fogel, 2002, 2005) – was missing. For Kichwa infants, however, these proximal ways of interaction were experienced as socially engaging, leading to the distinct response in infant gaze and positive affect. This interpretation is consistent with previous studies indicating that infants from independent milieus more frequently experience exclusive and dyadic attention, whereas caregivers in interdependent milieus tend to distribute their attention (Keller & Kärtner, 2013; LeVine, 1990). For example, mothers in rural Andean Peru have been reported to be very skilled at *muli-tasking*, such as washing clothes in the river and being sensitive towards the infant`s signals simultaneously (Fourment Sifuentes et al., 2021). In contrast to infants from Münster, Kichwa infants gazed longer at the experimenter during the baseline of the NTP than infants from Münster did, and they also showed a significant decrease in positive affect, which supports the argument that – for Kichwa infants – the baseline of the NTP also contained an *interactional component* in addition to the closeness and warmth established by body contact.

Overall, this study shows that the interruption of distal modes of communication, namely face-to-face interaction with synchronized voice, leads to similar responses across the two cultural milieus analyzed here. If the interaction is characterized by more proximal modes of communication, infants react in culture-specific ways. More specifically, the response is more pronounced in Kichwa infants, who usually experience more body contact. This particular finding supports the conclusion that infants come to expect, based on the everyday experience that they have, a certain pattern of stimulation and co-regulation that is functionally related to the development of a specific sense of self. Those results nicely complement previous findings from cross-cultural observational and behavioral studies on mother-infant interactions (Kärtner et al., 2010; LeVine, 1990; Wörmann et al., 2012, 2014), which reported culture-specific attentive and affective reactions in infants to a non-standardized social input.

### Limitations and future perspectives

A consequence of the larger project’s dense assessment plan, which consisted of weekly longitudinal assessments of mother-infant interactions, are relatively high drop-out rates, because rescheduling meetings could only rarely be realized. As a consequence, direct between-task or between-age comparisons of the same individuals could not be realized. Thus, in order to more directly compare still-face with no-touch effects, future studies should assess complete data from the same individuals, while balancing the order of tasks. Moreover, the reduction of the number of participants was greater in the Münster sample as compared to the Kichwa sample, leading to a greater reduction of power in the Münster sample.

We introduced the NTP as an ecologically valid variant of the classical SFP in cultures that emphasize more proximal modes of communication. In the present study, the NTP more closely resembles the average expected environment of Kichwa infants. Unfortunately, the direct comparison between tasks is limited, because facial expressions and gaze could not be coded during the second phase (i.e., close holding of the infant) of the baseline of the NTP. While future studies could ascertain a better visibility of infants’ communicative cues, future developments should also aim at further maximizing ecological validity of the NTP, for instance, by looking at proximal communication while caregivers carry their infants on their back.

Furthermore, one could look at responses beyond gaze and affect that might also mirror a cultural bias on distal modes of communication. For example, future studies could consider alternative indicators, which have successfully been used in previous still-face studies, such as physiological responses like heart rate or heart rate variability (Haley & Stansbury, 2003; Mesman et al., 2009). Furthermore, cross-cultural similarities and differences in infants’ responses across indicators could be tested vis-á-vis systematic variation in the intensity of stimulation within and across modalities, which might tell us more about the intensity and modal patterns of everyday social interactions and their consequences for infant communication. In each of the two paradigms the loss of a second, potentially important modality for mother-infant interaction is confounded with the loss of verbal/vocal communication. If one is interested in the specific role of vocal communication for the effects reported, the evidence reported is not conclusive and future studies would need to explicitly address this question. Finally, these hypotheses and the interpretation of these findings – coming from a standardized behavioral study in two cultural milieus – were based on specific assumptions concerning the everyday routines during caregiver-infant interaction that were derived from cross-cultural observational studies in other interdependent cultures, some of which were also from Indigenous American communities. Future studies should aim at grounding these assumptions in ecologically valid observations of caregiver-infant interactions from the same cultural communities.

## Conclusion

Complementing previous studies, we analyzed infants’ reactions to two standardized social interactions: the Still-Face (SFP) and the No-Touch Paradigm (NTP). Overall, our findings provide further evidence for the universality of the still-face effect, suggesting that – at 3 and 4.5 months of age – the threshold for infants across cultures to engage in face-to-face interactions is very low, leading to similar responses once this interaction is interrupted. The no-touch effect was more pronounced in the Kichwa ethnic group, which – along with their stronger decrease in gaze during the SFP at 3 months of age – supports the idea that infants have already internalized culture-specific interactional histories. More generally, the reactions of Kichwa infants during the NTP call attention to facets of early social interactions that have been neglected by researchers in WEIRD countries studying mainly WEIRD subjects.

## Appendix 1

### Effects of parity and gender on the still-face effect and the no-touch effect at 3 and 4.5 months

In the SFP, neither main effects of gender and parity nor their interactions with culture and phase were significant, when including them as an additional factor into separate ANOVAs.

In the NTP, the effects of phase, cultural milieu, and cultural milieu × phase of the three-factorial ANOVA were identical to the results of the two-factorial ANOVA reported in the Results section in all cases and in addition to that we found significant cultural milieu × gender and phase × parity interactions:

Regarding infant gaze at 3 months of age, the three-factorial ANOVA yielded a significant phase × parity interaction, *F*(1, 42) = 6.39, *p* = .015, *η*^2^ = .13. The effects of phase, *F*(1, 42) = 9.16, *p* = .004, *η*^2^ = .18, cultural milieu, *F*(1, 42) = 0.06, *p* = .20, *η*^2^ = .04, and cultural milieu × phase, *F*(1, 42) = 8.77, *p* = .005, *η*^2^ = .17, were identical to the results of the two-factorial ANOVA. The effect of parity and the interaction phase × culture × parity were not significant, *F*s < 0.74, *p*s > .398, *η*^2^s < .02. When including gender as an additional factor at 4.5 months of age, the three-factorial ANOVA yielded a significant cultural milieu × gender interaction, *F*(1, 40) = 4.16, *p* = .048, *η*^2^ = .09. The effects of phase, *F*(1, 40) = 4.06, *p* = .051, *η*^2^ =.09 , cultural milieu, *F*(1, 40) = 2.07, *p* =.158, *η*^2^ = .05, and cultural milieu × phase, *F*(1, 40) = 6.71, *p* = .013, *η*^2^ = .14, were identical to the results of the two-factorial ANOVA. The effect of gender and the interaction phase × culture × gender were not significant, *F*s < 0.74, *p*s > .392, *η*^2^s < .02.

With respect to positive affect at 3 months of age, the three-factorial ANOVA yielded a significant phase × parity interaction, *F*(1, 42) = 9.17, *p* = .004, *η*^2^ = .18. The effects of phase, *F*(1, 42) = 9.04, *p* = .004, *η*^2^ = .18, cultural milieu, *F*(1, 42) = 0.06, *p* = .811, *η*^2^ = .00, and cultural milieu × phase, *F*(1, 42) = 4.05, *p* = .051, *η*^2^ = .09, were identical to the results of the two-factorial ANOVA. The effect of parity and the interaction phase × cultural milieu × parity were not significant, *F*s < 2.44, *p*s > .128, *η*^2^s < .06. When including gender as an additional factor at 4.5 months of age, the three-factorial ANOVA yielded a significant cultural milieu × gender interaction, *F*(1, 40) = 6.82, *p* = .013, *η*^2^ = .15. The effects of phase, *F*(1, 40) = 12.29, *p* = .001, *η*^2^ = .24, cultural milieu, *F*(1, 40) = 0.06, *p* = .811, *η*^2^ = .00, and cultural milieu × phase, *F*(1, 40) = 4.31, *p* = .044, *η*^2^ = .10, were identical to the results of the two-factorial ANOVA. The main effect of gender and the interaction phase × cultural milieu × gender were not significant, *F*s < 0.50, *p*s > .478, *η*^2^s < .02.

## Appendix 2

### Attentional and affective reactions during the still-face and the no-touch paradigm at 3 and 4.5 months excluding immigrant mothers

Table 4

**Table 4:** Means and standard deviations for attentional and affective reactions during the still-face paradigm at 3 and 4.5 months excluding mothers with a migration background

	Baseline		Interruption phase		Partial η^2^		
	Münster	Kichwa	Münster	Kichwa	CM	P	CM × P
3 months	*M* (*SD*)	*M* (*SD*)	*M* (*SD*)	*M* (*SD*)			
Gaze [0-1]	.52 (.24)	.69 (.24)	.35 (.22)	.24 (.22)	.01	.65**	.26**
Positive affect [0-1]	.28 (.21)	.25 (.16)	.10 (.11)	.08 (.11)	.01	.49**	.00
Negative affect [0-1]	.09 (.13)	.07 (.11)	.16 (.29)	.13 (.19)	.01	.09	.00
4.5 months							
Gaze [0-1]	.44 (.29)	.50 (.28)	.25 (.19)	.21 (.18)	.00	.54**	.05
Positive affect [0-1]	.24 (.17)	.21 (.19)	.12 (.17)	.06 (.09)	.03	.34**	.01
Negative affect [0-1]	.02 (.04)	.03 (.05)	.10 (.16)	.11 (.16)	.01	.20**	.00

*Note*. Still-face effect at 3 months: *N* = 17 for the Münster sample and *N* = 24 for the Kichwa sample.

At 4.5 months: *N* = 16 for the Münster sample and *N* = 27 for the Kichwa sample.

* *p* < .05; ** *p* < .01

Table 5

**Table 5:** Means and Standard deviations for attentional and affective reactions during the no-touch paradigm at 3 and 4.5 months excluding mothers with a migration background

	Baseline		Interruption phase		Partial η^2^		
	Münster	Kichwa	Münster	Kichwa	CM	P	CM × P
3 months	*M* (*SD*)	*M* (*SD*)	*M* (*SD*)	*M* (*SD*)			
Gaze [0-1]	.37 (.28)	.59 (.31)	.33 (.26)	.29 (.28)	.03	.26**	.16**
Positive affect [0-1]	.15 (.18)	.21 (.21)	.13 (.18)	.05 (.09)	.00	.16**	.10*
Negative affect [0-1]	.06 (.10)	.06 (.10)	.24 (.29)	.22 (.28)	.00	.26**	.00
4.5 months							
Gaze [0-1]	.30 (.27)	.53 (.28)	.35 (.25)	.30 (.22)	.04	.08	.18**
Positive affect [0-1]	.19 (.26)	.24 (.22)	.13 (.18)	.07 (.10)	.00	.28**	.09
Negative affect [0-1]	.03 (.07)	.01 (.03)	.18 (.28)	.16 (.21)	.00	.31**	.00

*Note*. No-touch effect at 3 months: *N* = 17 for the Münster sample and *N* = 26 for the Kichwa sample. At 4.5 months: *N* = 15 for the Münster sample and *N* = 26 for the Kichwa sample.

* *p* < .05; ** *p* < .01

Table 6

**Table 6:** Means and standard deviations for attentional and affective reactions during the still-face paradigm at 3 and 4.5 months excluding mothers who migrated as adults to Germany

	Baseline		Interruption phase		Partial η2		
	Münster	Kichwa	Münster	Kichwa	CM	P	CM × P
3 months	*M* (*SD*)	*M* (*SD*)	*M* (*SD*)	*M* (*SD*)			
Gaze [0-1]	.54 (.25)	.69 (.24)	.36 (.22)	.24 (.22)	.00	.66**	.27**
Positive affect [0-1]	.28 (.20)	.25 (.16)	.10 (.11)	.08 (.11)	.01	.51**	.00
Negative affect [0-1]	.09 (.13)	.07 (.11)	.16 (.29)	.13 (.19)	.01	.09	.00
4.5 months							
Gaze [0-1]	.47 (.30)	.50 (.28)	.28 (.20)	.21 (.18)	.00	.55**	.05
Positive affect [0-1]	.23 (.16)	.21 (.19)	.11 (.16)	.06 (.09)	.02	.35**	.01
Negative affect [0-1]	.02 (.04)	.03 (.05)	.13 (.18)	.11 (.16)	.00	.24**	.01

*Note*. Still-face effect at 3 months: *N* = 18 for the Münster sample and *N* = 24 for the Kichwa sample.

At 4.5 months: *N* = 18 for the Münster sample and *N* = 27 for the Kichwa sample.

* *p* < .05; ** *p* < .01

Table 7

**Table 7:** Means and standard deviations for attentional and affective reactions during the no-touch paradigm at 3 and 4.5 months excluding mothers who migrated as adults to Germany

	Baseline		Interruption phase		Partial η2		
	Münster	Kichwa	Münster	Kichwa	CM	P	CM × P
3 months	*M* (*SD*)	*M* (*SD*)	*M* (*SD*)	*M* (*SD*)			
Gaze [0-1]	.36 (.28)	.59 (.31)	.33 (.25)	.29 (.28)	.04	.25**	.18**
Positive affect [0-1]	.16 (.18)	.21 (.21)	.13 (.18)	.05 (.09)	.00	.17**	.10*
Negative affect [0-1]	.06 (.10)	.06 (.10)	.23 (.28)	.22 (.28)	.00	.25**	.00
4.5 months							
Gaze [0-1]	.34 (.28)	.53 (.28)	.38 (.25)	.30 (.22)	.02	.10*	.18**
Positive affect [0-1]	.20 (.27)	.24 (.22)	.16 (.20)	.07 (.10)	.01	.26**	.12*
Negative affect [0-1]	.02 (.06)	.01 (.03)	.16 (.26)	.16 (.21)	.00	.30**	.00

*Note*. No-touch effect at 3 months: *N* = 18 for the Münster sample and *N* = 26 for the Kichwa sample. At 4.5 months: *N* = 17 for the Münster sample and *N* = 26 for the Kichwa sample.

* *p* < .05; ** *p* < .01

## Footnotes

1 Beyond their identification as Kichwas, many families near Otavalo call themselves *Kichwa-Otavalos* and families near Cotacachi specify their identification as *Kichwa-Cotacachis*. Hereafter, we use *Kichwa(s)* for Kichwa-Otavalo and Kichwa-Cotacachi families.

2 Based on transformed values, this effect was significant, *F*(1,42) = 5.34, *p* = .026, *η*^*2*^ = .11 .
